# Revisions of the genera *Lurama* Schaus, 1928 and *Ulmara* Schaus, 1928 (Lepidoptera, Mimallonoidea, Mimallonidae) with the descriptions of three new *Ulmara* species and a new genus

**DOI:** 10.3897/zookeys.611.9058

**Published:** 2016-08-15

**Authors:** Ryan A. St. Laurent

**Affiliations:** 1 McGuire Center for Lepidoptera and Biodiversity, Florida Museum of Natural History, University of Florida, 3215 Hull Road, Gainesville, FL 32611-2710 USA

**Keywords:** Andean, Bolivia, Colombia, Cunicumara anae, Ecuador, Peru, Ulmara azurula, Ulmara dombroskiei, Ulmara conjuncta

## Abstract

The Andean genera *Lurama* Schaus, 1928 and *Ulmara* Schaus, 1928 are revised. *Lurama* poses difficulty for revision due to lost male genitalia of the types of both described species. *Ulmara conjuncta* **sp. n.**, *Ulmara azurula* **sp. n.**, and *Ulmara dombroskiei* **sp. n.** are described as new in the genus *Ulmara*. A lectotype is designated for *Lurama quindiuna* Schaus, 1928 and *Ulmara rotunda* (Dognin, 1916). A new monotypic genus, *Cunicumara* **gen. n.**, which is externally similar to *Ulmara*, is described to include the new species *Cunicumara anae* **sp. n.** from low elevations of Bolivia and Paraguay. Male genital morphology does not support a close association of *Cunicumara* with *Lurama* or *Ulmara*. The latter two genera, however, are closely related based on similarities of male genitalia and biogeography.

## Introduction

Several recent works have been published focusing on the taxonomy of the previously understudied Mimallonidae, wherein 47 new species and four new genera have been described since 2012 (Herbin 2012, 2015, Herbin and Mielke 2014, Herbin and Monzón 2015, St Laurent and Dombroskie 2015, St Laurent and Dombroskie 2016, St Laurent and Mielke 2016, St Laurent and McCabe 2016). Each recent work since Herbin (2012) described new taxa of Mimallonidae from relatively low elevations (< 800 m) in Central America, South America, and the Caribbean, with relatively few taxa having been described from higher elevations. St Laurent and Dombroskie (2016) described two species apparently endemic to the Andean Cordillera Oriental from higher elevations, with the highest locale being about 1900 m. Until now, no work has been published focusing on Mimallonidae taxa endemic to higher elevations.

The present article revises two small and closely related genera, *Lurama* Schaus, 1928 and *Ulmara* Schaus, 1928, which are both endemic to high elevations (up to 3500 m) of the Andes, and describes an externally similar new, monotypic, genus from Bolivia and Paraguay. The genus *Lurama* currently consists of two species: *Lurama penia* (Dognin, 1919) and *Lurama quindiuna* Schaus, 1928. The monotypic genus *Ulmara* contains *Ulmara rotunda* (Dognin, 1916). Both genera are diagnosed based on external characters and male genitalia, and all currently known species are redescribed. The female of *Lurama quindiuna* is described and figured for the first time, and three new species are described in *Ulmara*.

## Methods

Dissections were performed as in Lafontaine (2004). St Laurent dissection preparation numbers follow the format of “St Laurent diss.:” followed by the preparation’s date, followed by a unique number for that date’s dissection. Morphological, including genitalia, terminology follows Kristensen (2003). The term “diaphragm” is used to describe the entirety of the membrane circumscribed by the XI segment ring (vinculum). Genitalia and abdomens are preserved in glycerol filled microcentrifuge tubes to preserve the three dimensional structure, or in the case of some dissections from MWM, are slide mounted.

Specimens from the following collections were examined:

AMNH
American Museum of Natural History, New York, New York, USA 


CMNH
Carnegie Museum of Natural History, Pittsburgh, Pennsylvania, USA 


CDH
 Personal collection of Daniel Herbin, Garidech, France 


CUIC
Cornell University Insect Collection, Ithaca, New York, USA 


MNHU
Museum für Naturkunde der Humboldt-Universität zu Berlin, Germany 


MWM
Museum Witt, Munich, Germany 


NHMUK
Natural History Museum, London, U.K. 


USNM
National Museum of Natural History [formerly United States National Museum], Washington, D.C., USA 


Figures were manipulated with Adobe Photoshop CS4 (Adobe 2008). Male genitalia are figured in natural color with CS4 “auto color” used to improve white backgrounds. Adult specimens and genitalia were photographed with a Macroscopic Solutions Macropod Pro and Canon EOS 6D DSLR camera body. Adult specimens were photographed with a Canon EF 100 Macro USM AF/MF Lens. Each specimen was photographed thirty times and the images were stacked using Zerene Stacking Software. However, the images of the Ecuadorian (Loja) female of *Lurama quindiuna*, (MWM), the female of *Ulmara rotunda*, (MNHU), and the Paraguayan paratype of *Cunicumara anae* sp. n., (CDH), were provided by their respective places of deposition, and thus did not undergo image stacking. A Macro Photo MP-E 65mm f/2.8 1–5× Manual Focus Lens for EOS was used to take thirty (3×) photographs of each genitalia preparation in ethanol under glass, and stacked using Zerene Stacking Software. Maps were created with SimpleMappr (Shorthouse 2010) and edited with CS4. All geographical coordinates are approximate, and are based on the localities provided on specimen labels. GPS data were acquired with Google Earth.

## Results and discussion

### Lurama

Taxon classificationAnimaliaLepidopteraMimallonidae

Schaus, 1928: 667

Luramana Strand, 1932: 147, unnecessary replacement name (Fletcher and Nye 1982).

#### Type species.

*Perophora penia* Dognin, 1919; Schaus 1928: 668, by original designation.

#### Diagnosis.

*Lurama* is immediately recognizable in both sexes among Mimallonidae by the yellow, yellow-gray, light-brown, or tan coloration of all wings, combined with prominent brown ante- and postmedial lines that strongly contrast against the lighter ground color. Wing veins are usually obvious and contrast against the ground color due to prominent brown scales lining them. Male genitalia are simple, but recognizable by the broad, short valves and the setae-covered lobes projecting from the transtilla. In *Lurama* the phallus is very narrow, pointed, and curved, being somewhat fishhook shaped. *Lurama* male genitalia are most similar to those of *Ulmara*, but can be distinguished by the following characters seen in *Lurama*: a much narrower phallus, less triangular valves, the absence of the gnathos, setae-covered structures on the ventrum of VIII, and teeth on the valves. The female genitalia are small, but stout and robust structures, with extremely reduced apophyses anteriores and corpus bursae. The highly reduced corpus bursae is the smallest in overall size in the family (compared to those examined so far). The female genitalia are similar in general structure to those of the female of *Ulmara*, but the genitalia of *Lurama* lack the broader, setae-covered lateral posterior lobe of VIII, and have a larger, though still very small by mimallonid standards, ductus bursae and corpus bursae, reflecting the much broader phallus of *Ulmara* relative to the extremely thin phallus of *Lurama*.

#### Description.

**Male.** *Head*: Tan brown to straw colored, eyes very large, occupying more than two thirds area of head, bordered posteriorly by dark scales; antenna coloration as for head, bipectinate to tip; labial palpus very reduced, three segmented, tufted ventrally, especially basal-most segment, palpus not extending beyond frons, heavily clothed in long scales, scales darker dorsally. *Thorax*: Coloration as for head, though it may be somewhat lighter, lustrous gold overall, darker-brown scales present on prothoracic collar. *Legs*: Coloration as for thorax, vestiture fine, appearing as spun gold. Tibial spurs somewhat elongated, tubular, clothed in scales except for tip. *Forewing dorsum*: Forewing length: 14.5–18 mm, wingspan: 27–35 mm, n=24. Variable overall; short, subtriangular to triangular, margin nearly straight from apex until after passing M_3_ where wing smoothly curves toward anal margin. Ground color variable from brown to tan to nearly yellow, overall lightly to heavily speckled by dark-brown or brown-gray petiolate scales, especially postmedially. Antemedial line brown, narrow to relatively wide, occasionally somewhat diffuse, slightly bowed outward. Postmedial line nearly straight or slightly curved inward from anal margin to Rs3, Rs4, or between Rs3 and Rs4, where line abruptly angled toward costa, forming nearly right angle, coloration and width as for antemedial line. Antemedial, medial, and postmedial areas concolorous, distance between lines variable. Costa and outer wing margin darker brown as in ante- and postmedial lines. Discal spot a dark-brown streak spanning width of discal cell, mesally slightly angled inward toward cell. Wing veins lined by dark-brown scales, colored as ante- and postmedial lines. Fringe poorly preserved (*Lurama penia*) or light brown with intermittent darker-brown scales. *Forewing ventrum*: Similar to dorsum but usually lighter; antemedial line nearly absent to absent and postmedial line may be lighter. *Hindwing dorsum*: Coloration as for forewing dorsum, antemedial line absent, postmedial line straight or curved outward. *Hindwing ventrum*: Following same pattern as forewing ventrum but postmedial line always curved outward. Base of wing usually covered by dark-brown scales from thorax. Frenulum present as single bristle, size somewhat variable. *Venation*: CuA_1_ arising nearly midway between lower angle of cell and CuA_2_; M_2_ and M_3_ arise from essentially same point of lower angle of cell, otherwise typical of Mimallonidae. *Abdomen*: Concolorous with thorax, distal tip with tuft of black scales. *Genitalia*: (Figs 11, 12) n=12. [based on *Lurama quindiuna* only] Simple; vinculum somewhat box-like, ventrally with reduced saccus, posterior edge of VIII attached to saccus, weakly bilobed. Uncus simple, triangular, excised laterally, highly truncated apically, laterally uncus beak-like. Gnathos absent. Valves short, irregularly shaped, somewhat triangular, truncated apically forming lobe, lobe occasionally narrow, valves often slightly curled mesally. Transtilla with inward angled lobes dorsal to phallus, from which extremely long, non-deciduous setae arise. Setae pointed outward directly over phallus. Saccular edge of valves with setae, occasionally nearly as long as those of lobes. Diaphragm forms balloon-like sac expanded inward into abdomen, sac covered in fine, inward facing setae surrounding phallus. Juxta partially fused to phallus, encircling it, lightly sclerotized, especially dorsal to phallus, weak sclerotization gives way to membrane contiguous with diaphragm setae-sac. Phallus short, very thin, tubular, bent, fishhook-like, terminal opening oblique, from which very small vesica emerges; base of phallus somewhat variable in length. **Female.** [based on *Lurama quindiuna* only] *Head*: As for male but antennae much smaller overall, pectination particularly shorter. *Thorax*: As for male though brown scales along prothoracic collar may be darker. *Legs*: As for male. *Forewing dorsum*: Forewing length: 17–18 mm, wingspan: 32–34 mm, n=2. As for male but slightly broader, longer, discal mark skewed toward M_3_. *Forewing ventrum*: Similar to dorsum but may be more suffused with brown, antemedial line absent. *Hindwing dorsum*: Coloration as for forewing dorsum, antemedial line absent, postmedial line straight or curved outward. *Hindwing ventrum*: Following same pattern as forewing ventrum but postmedial line always curved outward. Frenulum as multiple bristles, length shorter than in male. *Abdomen*: Concolorous with or slightly darker than thorax, distal tip with tuft of black scales. *Genitalia*: (Fig. 13) n=2. Stout, robust; tergite of VIII forms smooth, posteriorly directed arch, VIII heavily sclerotized laterally, forming posteriorly directed lobes covered in minute, fine setae. Apophyses anteriores highly reduced, nearly absent, apophyses posteriores elongate, spanning length of genitalia structure. Lamella antevaginalis wide, robust, concave, covered in short setae, ventral margin of lamella smoothly curved or slightly angled upward mesally. Ductus bursae short, very narrow. Corpus bursae highly atrophied, baglike, wing scales present within corpus bursae in both dissections. Base of papillae anales with nested accordion-like sclerotizations. Papillae anales somewhat rectangular laterally, dorsal corner may be somewhat extended as lobe; papillae anales covered with fine setae.

#### Remarks.

*Lurama* is a small, easily recognizable genus endemic to relatively high elevations of the Andes of Colombia and Ecuador. See individual species remarks for unresolvable issues pertaining to the type specimens of the two named species. A holistic revision is not possible for *Lurama* due to the issues pertaining to the type material of the named species; however, the genus is included in the present work due to the biogeographic similarity to *Ulmara* and the obvious homology in structures of the male and female genitalia. I recognize that more than one taxa likely exists under the name *Lurama quindiuna* in the present treatment, however due to aforementioned issues which will be explained in detail in each respective species remarks sections, no new taxa are described in *Lurama*.

#### Key to species of Lurama*

**Table:** 

1	Forewing rounded apically, ante- and postmedial lines broad, somewhat diffuse (Fig. 1)	***Lurama penia***
2	Forewing triangular, somewhat pointed apically, ante- and postmedial lines usually sharply defined (Figs 2–10)	***Lurama quindiuna***

*Note: the female of *Lurama penia* is unknown.

**Figure 1.**
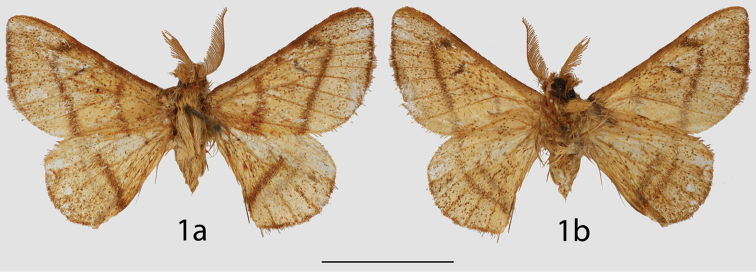
*Lurama penia* holotype ♂, **a** dorsal **b** ventral. Colombia, Cundinamarca/Distrito Capital, Bogotá, 2800–3200 m (USNM). Scale bar = 1 cm.

**Figures 2–6.**
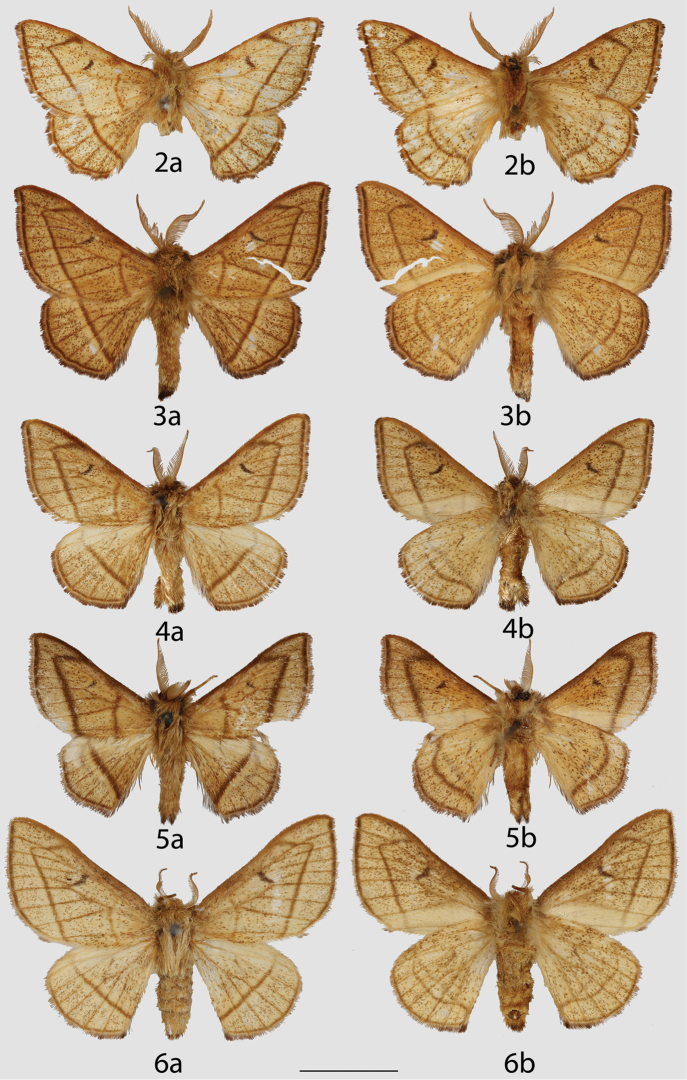
Colombian *Lurama quindiuna* *s. l.* adults, **a** dorsal **b** ventral. **2** Lectotype ♂, Quindío, Paso del Quindío, 3500 m (MNHU) **3** ♂, Cundinamarca, Bogotá, Pueblo Guasca (USNM) **4, 5** ♂, Specimens unlabeled, but almost certainly the same data as Figure 3 (USNM) **6** ♀, Unlabeled, but almost certainly the same data as Figures 3–5 (USNM). Scale bar = 1 cm.

**Figures 7–10.**
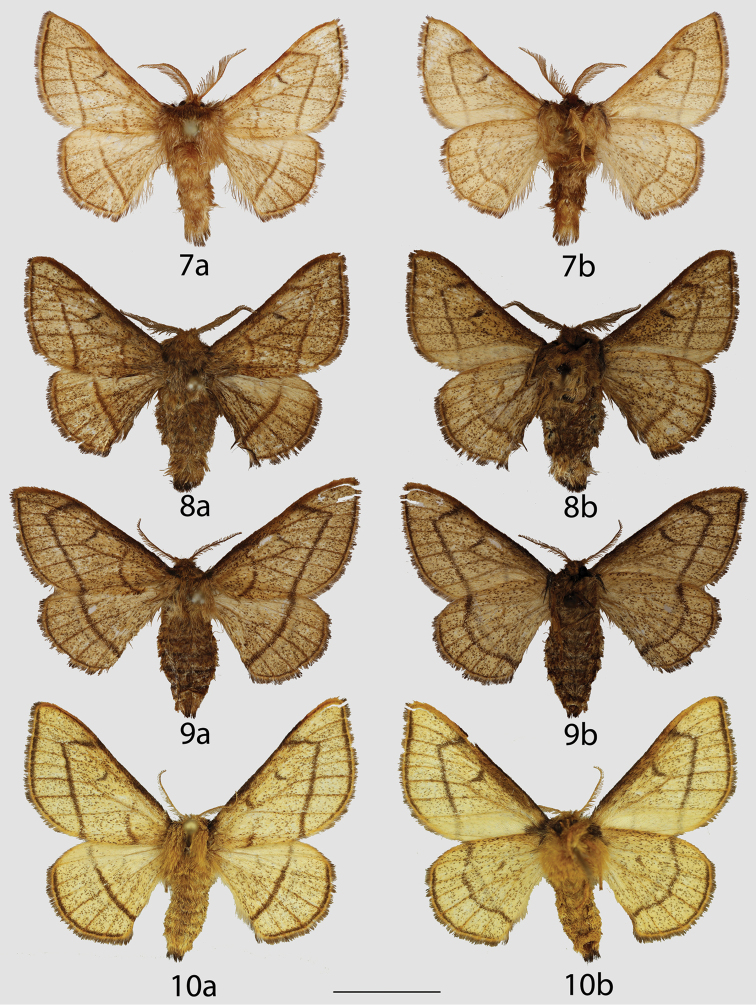
Ecuadorian *Lurama quindiuna* *s. l.* adults, **a** dorsal **b** ventral. **7** ♂, Carchi, 35 km W. of Tufinño, west slope, 3120 m (CMNH) **8** ♂, Napo, South Slopes, Cerro Sumaco, 2950 m (CMNH) **9** ♀, Napo, South Slopes, Cerro Sumaco, 2950 m (CMNH) **10** ♀, Loja, Road between Loja-Zamora, 2700 m [photo courtesy of Harald Sulak] (MWM). Scale bar = 1 cm.

### Lurama penia

Taxon classificationAnimaliaLepidopteraMimallonidae

(Dognin, 1919)

Fig. 1 ; Map 1

Perophora penia Dognin, 1919: 6, 7
Lurama penia ; Schaus 1928
Lurama penia ; Gaede 1931
Lurama penia ; Becker 1996

#### Type material.

**Holotype**, ♂. **COLOMBIA: Cundinamarca/Distrito Capital**: Bogota [Bogotá], Colombia, 28–3200 m, Coll. Fassl / Dognin, Collection/ *Perophora penia*, type ♂ Dgn./ ♂ genitalia slide. 6 June ’28, C.H. #5. [genitalia prep. not located]/ [label with wing vein sketch]/ [Holo]Type No.: 29698 U.S.N.M./ USNM-Mimal: 1108/ (USNM, examined). Type locality: Colombia: Cundinamarca/Distrito Capital: Bogotá.

#### Diagnosis.

*Lurama penia* can be distinguished from *Lurama quindiuna* by the smaller size, shorter, more rounded wings, and somewhat more diffuse, broader postmedial and antemedial lines on the wings. The lines are also closer together and farther from the wing margin than in *Lurama quindiuna*. Additionally, the forewing postmedial line has its apical angle intersecting Rs4 very near the fork of Rs3+Rs4, rather than being much more distant from this fork in the other species.

#### Description.

**Male.** *Head*: As for genus but tan brown. *Thorax*: Coloration as for head, darker-brown scales present on prothorax. *Legs*: Coloration as for thorax, poorly preserved. *Forewing dorsum*: Forewing length: 14.5 mm, wingspan: ~27 mm, n = 1. Short, vaguely triangular, margin nearly straight from apex until after passing M_3_ where wing smoothly curves toward anal margin. Ground color yellowish tan, overall lightly speckled by dark petiolate scales, especially postmedially. Antemedial line brown, relatively wide, somewhat diffuse, slightly curving outward. Postmedial line nearly straight from anal margin to Rs4 where it abruptly angles toward costa perpendicularly, coloration and width as for antemedial line. Antemedial, medial, and postmedial areas concolorous. Costa and outer wing margin darker brown. Discal spot a dark streak spanning width of discal cell, mesally slightly angled inward toward cell. *Forewing ventrum*: Similar to dorsum but with more concentrated speckling postmedially; antemedial line nearly absent, postmedial line fainter. *Hindwing dorsum*: Coloration as for forewing dorsum, antemedial line absent, postmedial line not reaching anterior wing margin. *Hindwing ventrum*: Following same pattern as forewing ventrum but postmedial line curved outward rather than straight as on dorsum. *Abdomen*: Partially missing, but anterior segments concolorous with thorax. *Genitalia*: Not examined. **Female.** Unknown.

#### Distribution

**Map 1.**
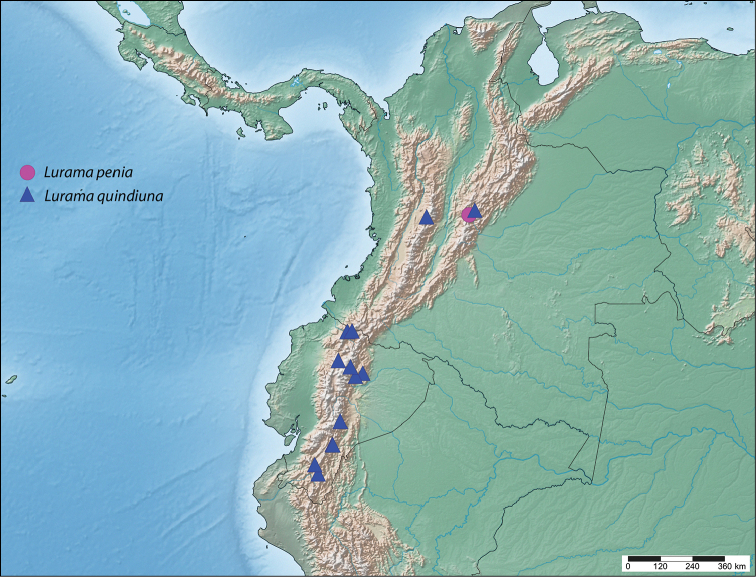
Known distribution of *Lurama*.

**(Map 1).** *Lurama penia* is known only from the holotype, collected between 2800 and 3200 m near Bogotá, Colombia.

#### Remarks.

This species is apparently very rarely collected, as it is known only from the male holotype. Unfortunately, the genitalia preparation of the holotype, probably made by Carl Heinrich (R. Hutchings pers. comm.), is lost and could not be located by the individuals tasked with trying to find it at the USNM. Schaus (1928) compared the genitalia of *Lurama* to that of *Ulmara*, and considered them similar. I note that among Schaus’s examined *Lurama* material at the USNM and MNHU, the only dissected male *Lurama* specimen is the holotype of *Lurama penia*; therefore it is reasonable to assume that the missing genitalia of *Lurama penia* are similar to those of *Ulmara* because apparently Schaus did not look at any other *Lurama* dissections. This is expected given the similarity between *Ulmara* male genitalia and those of *Lurama quindiuna*, for which the male genitalia are available for examination.

As for *Lurama quindiuna* below, the issue of missing type genitalia for these two species results in some difficulties determining the identity of non-type specimens. Fortunately, external diagnostic characteristics are ample enough to maintain both currently described species as valid, but prevents me from describing new species in the genus at this time. It is vital that more material be located for this and the following species at each of their respective type localities so that more conclusive diagnostic characters can be given, particularly in regards to the genitalia.

### Lurama quindiuna

Taxon classificationAnimaliaLepidopteraMimallonidae

Schaus, 1928, sensu lato

Figs 2–6 , 7–10 , 11–12 , 13 ; Map 1

Lurama quindiuna Schaus, 1928: 669; Fig. ♂ 88h
Lurama quindiuna ; Gaede 1931

#### Type material.

**Lectotype (here designated)**, ♂. **COLOMBIA: Quindío**: Typus/ Paso del Quindiu, Colomb. Cent. Cord. [Paso del Quindío, Cordillera Central], 3500 m, Coll. Fassl/ *Lurama quindiuna*, type Schaus/ St Laurent diss.: 2-14-16:1 [abdomen and genitalia excluded from holotype, see remarks]/ LECTOTYPE ♂ *Lurama quindiuna* designated by St Laurent, 2016 [handwritten red label]/ (MNHU). Type locality: Colombia: Paso del Quindío.

#### Additional specimens examined.

(60 ♂, 3 ♀) **COLOMBIA: Cundinamarca**: 7 ♂, Bogotá, Puebla Guasca: F. Johnson donor, USNM-Mimal: 2640–2643, 2645, 2646, St Laurent diss.: 2-14-16:3, 2-14-16:4, 2-14-16:5, 2-19-16:1 one specimen labeled as “*Lurama quindiuna* nr topotype?” by Schaus (6 ♂, USNM); (1 ♂, CUIC). 9 ♂, 1 ♀, No locality labels, but almost certainly belonging to F. Johnson’s series from Bogotá, Puebla Guasca, USNM-Mimal: 2866, 2879–2886, 2888, St Laurent diss.: 3-4-16:1, 3-4-16:2, 3-4-16:3, the following label is hereby added to these 10 specimens: “Originally unlabeled, but almost certainly: COLOMBIA: Cudinamarca: Puebla Guasca nr. Bogotá ex. F. Johnson [locale det.: R. St Laurent]” (USNM). **ECUADOR: Carchi**: 3 ♂, 35 km W. Tufiño, west slope, cloud forest, 3120 m: 20.XI.1987, R. Davidson, C. Young, St Laurent diss.: 3-11-16:3 (CMNH). 5 ♂, El Angel Ecol. Reserve, road Tulcan – El Chical, 0°48'46"N, 78°00'40"W, 3300 m: 14.XI.2012, leg. Victor Sinyaev, Expedition Ron Brechlin (MWM). 5 ♂, El Angel Ecol. Reserve, 0°45'31"N, 78°01'40"W, 3320 m: 7–8.XI.2012, leg. Victor Sinyaev, Expedition Ron Brechlin (MWM). 1 ♂, El Moran, 0°45'50"N, 78°02'38"W, 2940 m: 1–3.V.2012, leg. R. Brechlin & V. Siniaev (MWM). **Pichincha**: 1 ♂, Oyacachi, montane tropical forest, 3300 m: Jan Hillman, St Laurent diss.: 3-14-16:2 (CMNH). **Napo**: 2 ♂, 1 ♀, South Slopes, Cerro Sumaco, wet cloud/moss forest, 2950 m: 18.XI.1995, Jan Hillman, St Laurent diss.: 3-14-16:4, 3-14-16:5 (CMNH). 2 ♂, Cordillera Huacamayos [Cordillera Guacamayos], Estero Chico, virgin humid forest, 2650 m: 5.VIII.1996, J. Hillman, St Laurent diss.: 3-11-16:2, 3-14-16:3 (CMNH). 1 ♂, 10 km E. Papallacta, Hacienda Bosque on Quito-Baeza road, disturbed montane forest, 2600 m: 11.XI.1995, Jan Hillman (CMNH). 3 ♂, Papallacta, Rio San Pedro, 0°22'56"S, 78°7'27"W, 3010 m: 18.I.2012, 22.III.2012, leg. R. Brechlin & V. Siniaev (MWM). 2 ♂, Rio Papallacta, Cuyuja, 0°25'17"S, 78°1'19"W, 2525 m: 6.XI.2011, leg. V. Siniaev & O. Romanoc (MWM). **Azuay**: 1 ♂, Road between Gualaceo-Méndez, Limon, 2°59'4"S, 78°39'50"W, 3410 m: 8.III.2013, leg. Ackermann, Käch, & Dr. R. Brechlin (MWM). **Loja**: 1 ♀, Road Loja-Zamora, 3°58'45"S, 79°08'28"W, 2700 m: 22.II.2012, leg. R. Brechlin & V. Siniaev (MWM). 2 ♂, 6 km S Saraguro, 3°40'01"S, 79°15'17"W, 3065 m: 20.II.2012, leg. R. Brechlin & V. Siniaev (MWM). 1 ♂, 15 km E Loja to Zamora, 3°58'45"S, 79°08'28"W, 2700 m: 1.III.2011, leg. H. Kaech & R. Brechlin (MWM). **Morona-Santiago**: 2 ♂, Road Gualaceo, Plan de Milagro, 3°00'13"S, 78°38'46"W, 3176 m: 19.XI.2011, leg. V. Siniaev & O. Romanov (MWM); 17.II.2012, leg. R. Brechlin & V. Siniaev (MWM). 3 ♂, 30 km Road Plan de Milagro to Gualaceo, 3°00'21"S, 78°38'28"W, 2970 m: 1–2.II.2012, leg. R. Brechlin & V. Siniaev (MWM). 1 ♂, 62 km road Rio Bamba-Macas, 2°12'40"S, 78°23'51"W, 2700 m: 27.III.2012, leg. R. Brechlin & V. Siniaev, Genital präparat Nr. 28.995 Museum Witt München (MWM). 7 ♂, 34 km Road Plan de Milagro to Gualaceo, 3°00'13"S, 78°38'46"W, 3176 m: 30.I.2012, leg. R. Brechlin & V. Siniaev (MWM). 2 ♂, 34 km Road Plan de Milagro to Gualaceo, 3°01'24"S, 78°35'6"W, 2157 m, 28.I.2012, leg. R. Brechlin & V. Siniaev (MWM).

#### Diagnosis.

*Lurama quindiuna* can be distinguished from the previous species by the larger size, more triangular wings, and by the (usually) thinner, more well-defined ante- and postmedial lines. The lines are situated closer to the wing margin than in *Lurama penia*, and form a sharp angle apically. Additionally, the forewing postmedial line has its apical angle either intersecting Rs4 or between Rs3 and Rs4 and is distant from the fork of Rs3+Rs4.

#### Description.

**Male.** *Head*: As for genus. *Thorax*: As for genus. *Legs*: As for genus. *Forewing dorsum*: Forewing length: 14.5–17.5 mm, avg.: 16.1 mm, wingspan: 27–35 mm, n=23. Somewhat variable; usually triangular, margin nearly straight from apex until after passing M_3_ where wing smoothly curves toward anal margin. Ground color variable from brown to tan to nearly yellow, overall lightly to heavily speckled by dark-brown or brown-gray petiolate scales, especially postmedially. Antemedial line brown, usually narrow, rarely wide, slightly bowed outward. Postmedial line nearly straight or slightly curved inward from anal margin to Rs3, Rs4, or between Rs3 and Rs4, where line abruptly angled toward costa, forming nearly right angle, coloration and width as for antemedial line. Antemedial, medial, and postmedial areas concolorous, distance between lines variable. Costa and outer wing margin darker brown as in ante- and postmedial lines. Discal spot a dark-brown streak spanning width of discal cell, mesally slightly angled inward toward cell. Wing veins lined by dark-brown scales, colored as for ante- and postmedial lines. Fringe light brown with intermittent darker- brown scales. *Forewing ventrum*: Similar to dorsum but usually lighter; antemedial line nearly absent to absent and postmedial line may be lighter. *Hindwing dorsum*: Coloration as for forewing dorsum, antemedial line absent, postmedial line straight or curved outward. *Hindwing ventrum*: Following same pattern as forewing ventrum but postmedial line always curved outward. Base of wing usually covered by dark-brown scales from thorax. Frenulum present as single bristle, size somewhat variable. *Abdomen*: Concolorous with thorax, distal tip with tuft of black scales. *Genitalia*: See generic description. **Female.** See generic description.

**Figures 11–12.**
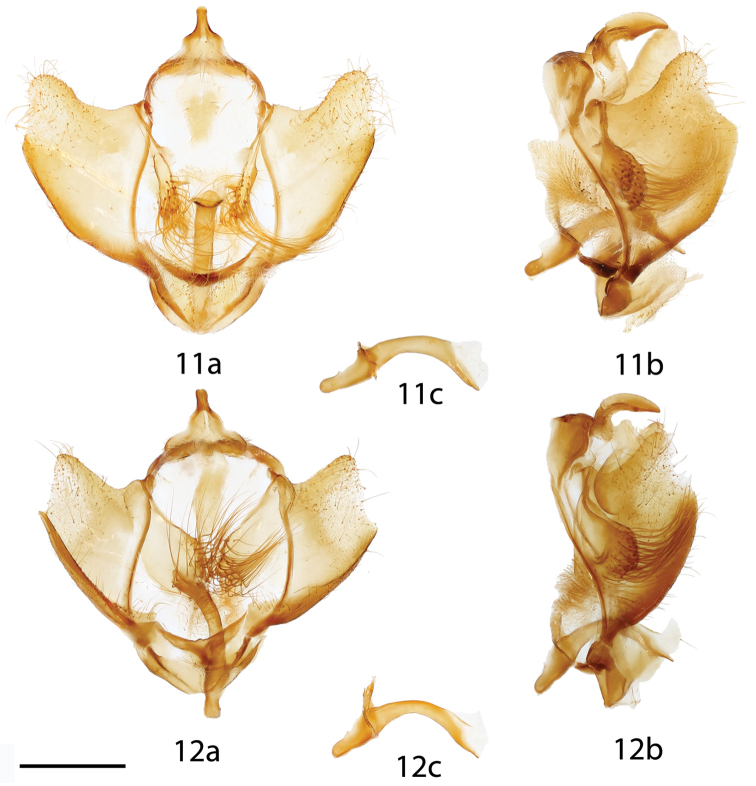
*Lurama quindiuna* ♂ genitalia, **a** ventral **b** lateral **c** phallus. **11** Colombia, Cundinamarca, Bogotá, Pueblo Guasca, St Laurent diss.: 2-14-16:5 (USNM) **12** Ecuador, Napo, Cordillera Guacamayos, Estero Chico, 2650 m, St Laurent diss.: 3-14-16:3 [note, phallus with remnant of juxta still attached] (CMNH). Scale bar = 1 mm.

**Figure 13.**
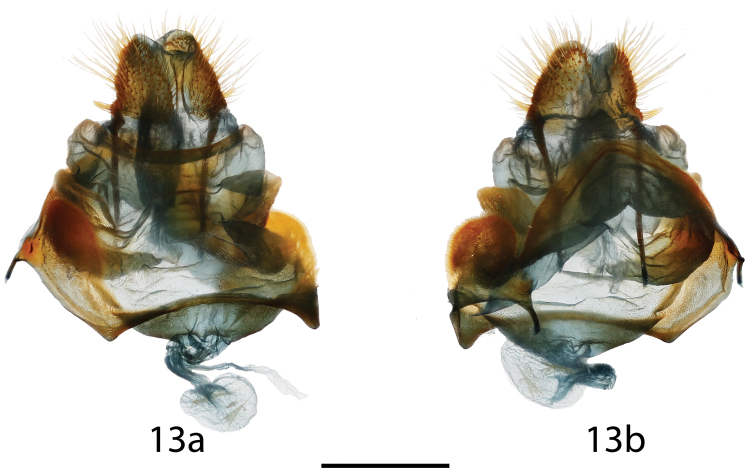
*Lurama quindiuna* ♀ genitalia, **a** ventral **b** dorsal. Specimen unlabeled, but almost certainly: Colombia, Cundinamarca, Bogotá, Pueblo Guasca, St Laurent diss.: 4-14-16:1 (USNM). Scale bar = 1 mm.

#### Distribution

**(Map 1).** *Lurama quindiuna* is an Andean species known from Colombia and Ecuador at moderate elevations ranging from 2157–3500 m. According to data on Ecuadorian specimens, this species can be encountered in humid montane cloud and moss forests.

#### Remarks.

Confusion surrounds the whereabouts of the genitalia of the lectotype of *Lurama quindiuna*. When initially examining the lectotype, I noticed that the abdomen was visibly small and noticeably lighter in color relative to the rest of the specimen. The abdomen was odd enough that it appeared out of place on the specimen, and a dissection further supported this notion. The dissection revealed complicated genitalia unlike any other in Mimallonidae, especially compared with other dissections of *Lurama quindiuna* from nearby locations. The elongated saccus and thin, golf club like phallus is reminiscent of some Bombycidae (pers. obs.), whereas the valve structure is similar to some Notodontidae. Alexander Schintlmeister (pers. comm.) has suggested Lasiocampidae rather than Notodontidae and Daniel Herbin (pers. comm.) mentioned the possibility of it belonging to Phiditiidae although Joël Minet (pers. comm.) does not consider this likely due to the presence of a saccus. So far, the exact determination of the genitalia is inconclusive. Apparently the abdomen of the *Lurama quindiuna* lectotype was misplaced and incorrectly replaced with a foreign abdomen at some point after its original description. Wolfram Mey (pers. comm.) confirmed that this, unfortunately, is a known issue with material in the MNHU. Furthermore, it is worth noting that Schaus (1928) figures the lectotype of *Lurama quindiuna*, illustrating a regularly proportioned and colored abdomen. Unfortunately, Schaus (1928) makes no mention of the abdomen nor genitalia in his original description. I chose not to figure these “mystery” genitalia for fear of causing inadvertent association with *Lurama*.

By Article 73.1.5, the abdomen (and my subsequent genitalia preparation: St Laurent diss.: 2-14-16:1) is hereby excluded from the lectotype of *Lurama quindiuna*, and although it will be preserved for historical purposes, it cannot be considered a component of this lectotype (ICZN 1999).

The lectotype of *Lurama quindiuna* differs in maculation from the series from Bogotá, particularly in regards to the arrangement of the forewing postmedial line. In the lectotype, the postmedial line is farther from the wing margin, angled toward the costa more proximal to the fork of Rs3 + Rs4, and has an apical angle that intersects Rs4 rather than being situated between Rs3 and Rs4. Furthermore, the postmedial and antemedial lines are closer together in the lectotype. While these characters were nearly consistently different between the lectotype and all specimens from Bogotá, Pueblo Guasca, substantial variations in these same characters were found within the Pueblo Guasca series (compare Figs 2–6). The variation within this series prevents me from considering these two populations as distinct, especially without access to genitalia of topotypical material. The material from Ecuador is also quite variable in the same characters, and thus there seems to be a high degree of phenotypic plasticity in the species, but I found no external characters applicable to any single geographic region. Male genitalia are rather consistent among all populations, with only minor variation in the valves, namely in their width, thickness, and curvature, as well as in the shape of the apical lobe. These minor variations are seen within both Colombian and Ecuadorian populations, but some Ecuadorian specimens tend to have thinner (in terms of thickness, not width), more concave valves. Antennal size also differs somewhat within populations, but more markedly between Colombian and Ecuadorian specimens such that Ecuadorian specimens tend to have shorter pectinations.

Female genitalia, however, do differ between Colombian and Ecuadorian *Lurama quindiuna* specimens. The width of the VIII tergite, shape of papillae anales and lamella antevaginalis, as well as the size of the corpus bursae all differed between these locations. However, given the lack of differentiating characters in the usually much more reliable male genitalia (pers. obs.) as well as the low sample size of female specimens, it does not seem reasonable to consider the Colombian and Ecuadorian populations separate species based on this information alone. Molecular evidence may later be useful in comparing these two populations, although recently collected Colombian material is currently lacking.

It is critical to locate more material of *Lurama quindiuna* from the type locality at Paso del Quindío, Colombia, so that the necessary genitalia comparisons between topotypical and other populations can be made. Also, it would be important to determine if populations from Paso del Quindío display similar levels of variation as in the other examined populations to further justify conclusions presented in the present work.

*Lurama quindiuna* was omitted from Becker (1996).

### Ulmara

Taxon classificationAnimaliaLepidopteraMimallonidae

Schaus, 1928: 666

#### Type species.

*Cicinnus rotunda* Dognin, 1916; Schaus 1928: 666, by original designation.

#### Diagnosis.

The unique genus *Ulmara* shows no apparent external resemblance to any known Mimallonidae. The stout, broad wings, dark, nearly black coloration, serrated postmedial lines, combined with impressively long pectinations of the antennae in males, are all characters that should immediately allow the recognition of *Ulmara*. Although the long pectinations of the antennae are seen in the other two genera treated in the present work, neither genus is so darkly colored. *Cunicumara* gen. n., described below, is similar in having both broad antennae and stout wings, but is easily distinguished by the straight, rather than serrate postmedial line present in *Ulmara*. The male genitalia of *Ulmara* are actually rather similar to those of *Lurama* (see diagnosis of *Lurama*) but wholly unlike those of *Cunicumara* gen. n. The genitalia of *Ulmara* are recognized by the short, broad phallus with a scoop-like ventral projection, apically-toothed valves, and the unique, paired, setae-covered sclerotizations on the ventrum of VIII, which are variously connected to an extension of the saccus.

#### Description.

**Male.** *Head*: Very dark brown to nearly black, eyes very large, occupying more than two-thirds area of head; antennal coloration pale tan to dark brown, if lighter in color, contrasting against dark head, scape with contrasting pale off-white tuft of scales, antenna bipectinate to tip, pectinations very long, the longest nearly one-fourth length of antenna overall; labial palpus relatively long, extending beyond head, three segmented, tufted ventrally, heavily clothed in long scales. *Thorax*: Coloration as for head but often with pale-gray to brown scales beneath darker-black to steel-blue scales, vestiture very long, shaggy, scales thin, scales of prothorax lighter brown. *Legs*: Coloration as for thorax, but lighter ventrally, vestiture fine, elongated. Femur and tibia clothed in particularly long scales. Tibial spurs somewhat elongated, tubular, clothed in light khaki-colored scales except for naked tip. *Forewing dorsum*: Forewing length: 17.5–21 mm, wingspan: 34–43 mm, n=16. Short subtriangular, margin convex, apex barely accentuated. Ground color usually very dark grayish black, often with nearly steel-blue sheen, older and worn specimens expose lighter pale-brown scales beneath thicker, darker-scales; wider petiolate scales absent. Antemedial line brown, zigzagged, usually obscured by darker surrounding scales so line nearly absent. Brown postmedial line serrated at each wing vein, postmedial line may be very distinct, especially when lighter scales present along inner side, and near tornus, or postmedial line may be overshadowed by darker surrounding scales; line angled toward costa at Rs4. Between Rs4 and costa, line slightly undulated, but otherwise less serrate, may be notched near costa. Location of postmedial line variable, from one-fourth wing length distant from wing margin to nearly two-thirds distant. Antemedial, medial, and postmedial areas concolorous, although postmedial area may be lighter gray blue than medial area, particularly along postmedial line. Discal spot a small white mark, either somewhat circular in shape or oblong. Fringe consisting of elongated scales, coloration as for medial area except some lighter off-white scales present in semi-regular pattern along margin. *Forewing ventrum*: Similar to dorsum but much lighter, grayer, maculation reduced; antemedial line absent, postmedial line less distinct to nearly absent, brown outline lacking if present on dorsum. *Hindwing dorsum*: Coloration as for forewing dorsum, antemedial line absent, discal mark absent, outer margin of wing may be serrate or mostly smooth. *Hindwing ventrum*: Following same pattern as forewing ventrum. Base of wing sometimes covered by dark-brown scales emanating outward from thorax. Frenulum present as single bristle. *Venation*: Similar to *Lurama* but CuA_1_ arises nearer to lower angle of cell; M_2_ and M_3_ do not originate from same point at lower angle of cell, and are more separated. *Abdomen*: Concolorous with thorax, but usually slightly lighter brown, distal tip with paired tuft of elongate scales. *Genitalia*: Simple; vinculum somewhat box-like or ovoid, ventrally with reduced saccus, projection of saccus attached to VIII, forming paired, setae-covered sclerotizations. Uncus simple, highly truncated apically with triangular base or broad basally, with reduced apical extension, laterally uncus beak-like. Gnathos variable, from nearly absent, to mesally gapped, to a single fused plate. Valves short, generally triangular, somewhat truncated apically, with single tooth projecting from saccular edge near apex of valve, tooth variable in length. Transtilla with weakly sclerotized, inward-facing setae-covered extensions, setae variable in thickness from very fine to thick. Setae pointed outward directly over phallus. Diaphragm forms small, balloon-like sac expanded inward into abdomen, sac covered in fine, inward-facing setae surrounding phallus. Juxta partially fused to phallus, encircling it, lightly sclerotized, especially dorsal to phallus. Phallus short, stout, anterior half tubular or bent, apex more heavily sclerotized, forming scoop-like extension below vesica; vesica small, sac-like, weakly scobinate, poorly differentiated diverticula may be present; base of phallus much narrower than apical half, but variable in thickness, angled backward from apex of phallus. **Female.** *Head*: As for male, but antennae much smaller overall, pectinations particularly shorter, labial palpus slightly reduced. *Thorax*: As for male. *Legs*: As for male, but vestiture seemingly sparser. *Forewing dorsum*: Forewing length: 21.5 mm, wingspan: 41 mm, n=2. As for male but more elongated, slightly narrower, discal mark reduced. *Forewing ventrum*: Similar to dorsum but much lighter, grayer, maculation reduced; antemedial line absent, postmedial line less distinct. *Hindwing dorsum*: Coloration as for forewing dorsum, antemedial line absent. *Hindwing ventrum*: Following same pattern as forewing ventrum. Frenulum as multiple bristles, length shorter than in male. *Abdomen*: Concolorous with thorax, similar to male but not truncated to a point. *Genitalia*: (Fig. 28) n=1. [based on *Ulmara conjuncta* sp. n. only] Stout; tergite of VIII forms smooth, thick posteriorly directed arch, laterally VIII heavily sclerotized, forming pair of posteriorly directed lobes on each side, anterior lobe covered in minute, fine setae, posterior lobe with about 10 sparse, long setae. Apophyses anteriores slightly more than half length of apophyses posteriores, apophyses posteriores very long, as long as length of genitalia structure. Lamella antevaginalis wide, concave, only posterior edge well sclerotized, covered in minute setae, ventral margin of lamella smoothly curved. Ductus bursae short, almost as wide as corpus bursae. Corpus bursae reduced, baglike. Papillae anales somewhat convex laterally, papillae anales covered with fine setae, setae at base of papillae anales more compactly distributed, forming lateral tuft.

#### Remarks.

The genus *Ulmara* contains some of the darkest colored and highest elevation inhabiting Mimallonidae, and is immediately recognizable by characters given in the diagnosis. Most species in the genus are quite similar to one another, but can be primarily differentiated by male genitalia. Distribution and overall size should also be adequate for simple diagnosis.

#### Key to species of Ulmara*

**Table:** 

1	Forewing length 19 mm or greater, usually not strongly iridescent blue (Figs 14–19), Colombia to Ecuador	**2**
–	Forewing length usually less than 19 mm, strongly iridescent (Figs 20–22), Peru southward	**3**
2	Ground coloration dark brown to blackish brown, postmedial line obvious due to brown inner lining (Figs 14–16), gnathos with mesal gap, phallus broad, not bent (Figs 23, 24). Colombia	***Ulmara rotunda***
–	Ground coloration almost black, postmedial line indistinct, no brown inner lining along postmedial line (Figs 17–19), gnathos fused mesally, phallus bent downward (Fig. 25). Ecuador	***Ulmara conjuncta* sp. n.**
3	Postmedial line one-quarter from wing margin (Fig. 20), gnathos highly reduced, setae on flaps extending from transtilla very thick, splayed saccular projections wider than vinculum, phallus narrow when viewed dorsally (Fig. 26)	***Ulmara azurula* sp. n.**
–	Postmedial line one-quarter to one-third from wing margin (Fig. 21, 22), gnathos reduced but almost converges mesally, setae on flaps extending from transtilla very thin, splayed saccular projections barely wider than vinculum, phallus broad, compressed, when viewed dorsally (Fig. 27)	***Ulmara dombroskiei* sp. n.**

*Note: the females of *Ulmara azurula* sp. n. and *Ulmara dombroskiei* sp. n. are unknown.

**Figures 14–16.**
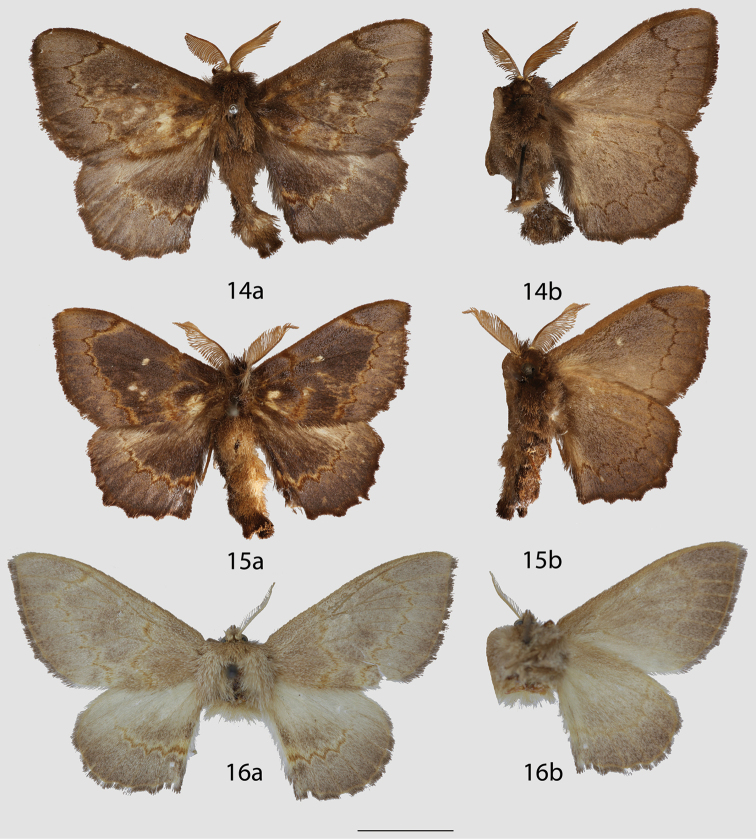
*Ulmara rotunda* adults, **a** dorsal **b** ventral. **14** Lectotype ♂, Colombia, Tolima, Nevado del Tolima, 3200 m (USNM) **15** ♂, Colombia, Tolima, Nevado del Tolima, 3200 m (MNHU) **16** ♀, Colombia, Cundinamarca/ Distrito Capital, Bogotá [photo courtesy of Wolfram Mey] (MNHU). Scale bar = 1 cm.

**Figures 17–19.**
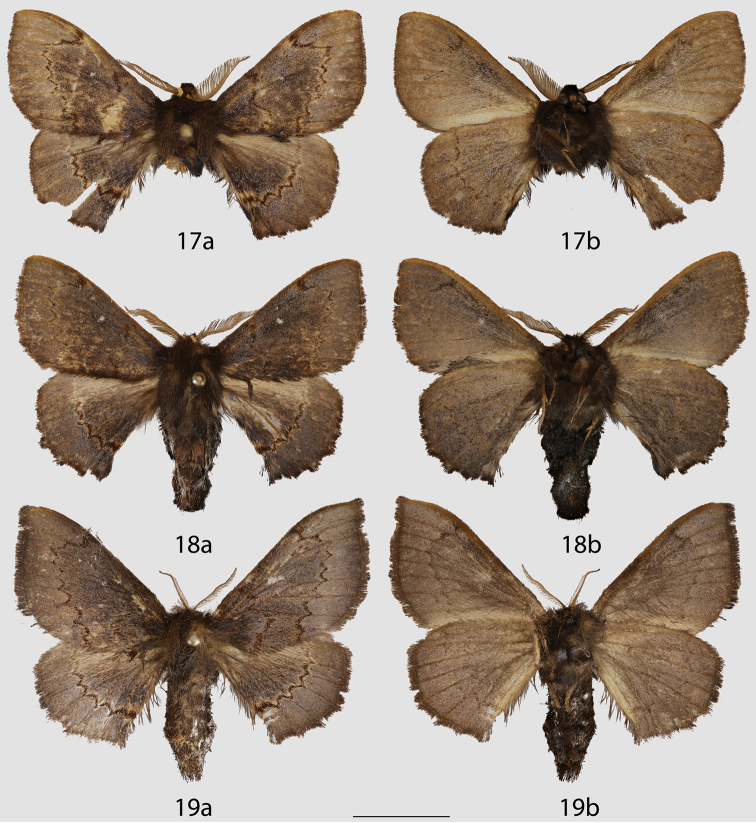
*Ulmara conjuncta* adults, **a** dorsal **b** ventral. **17** Holotype ♂, Ecuador, Loja, N. Loja, road to Cuenca, 2220 m (CMNH) **18** Paratype ♂, Ecuador, Napo, Cordillera Guacamayos, Estero Chico, 2650 m (CMNH) **19** Paratype ♀, Ecuador, Loja, Road between Loja-Zamora, 2700 m (MWM). Scale bar = 1 cm.

**Figures 20–22.**
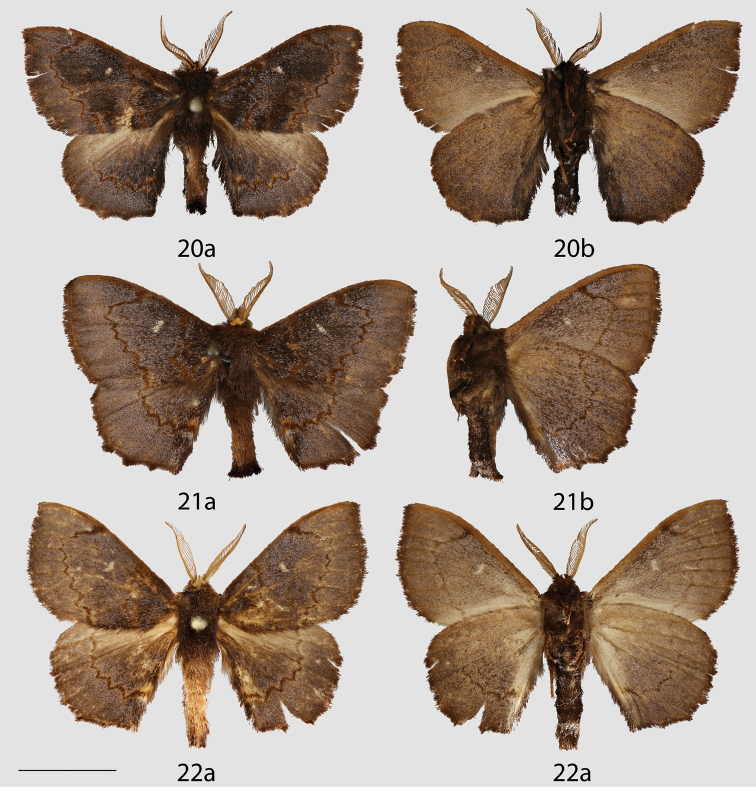
Peruvian *Ulmara* species adults, **a** dorsal **b** ventral. **20** *Ulmara azurula* holotype ♂, Peru, Huánuco, Carpish, 2700 m (AMNH) **21** *Ulmara dombroskiei* holotype ♂, Peru, Puno, Carabaya, Agualani, 9000 ft (NHMUK) **22** *Ulmara dombroskiei* paratype ♂, Peru, Puno, Carabaya, Santo Domingo (NHMUK). Scale bar = 1 cm.

**Figures 23, 24.**
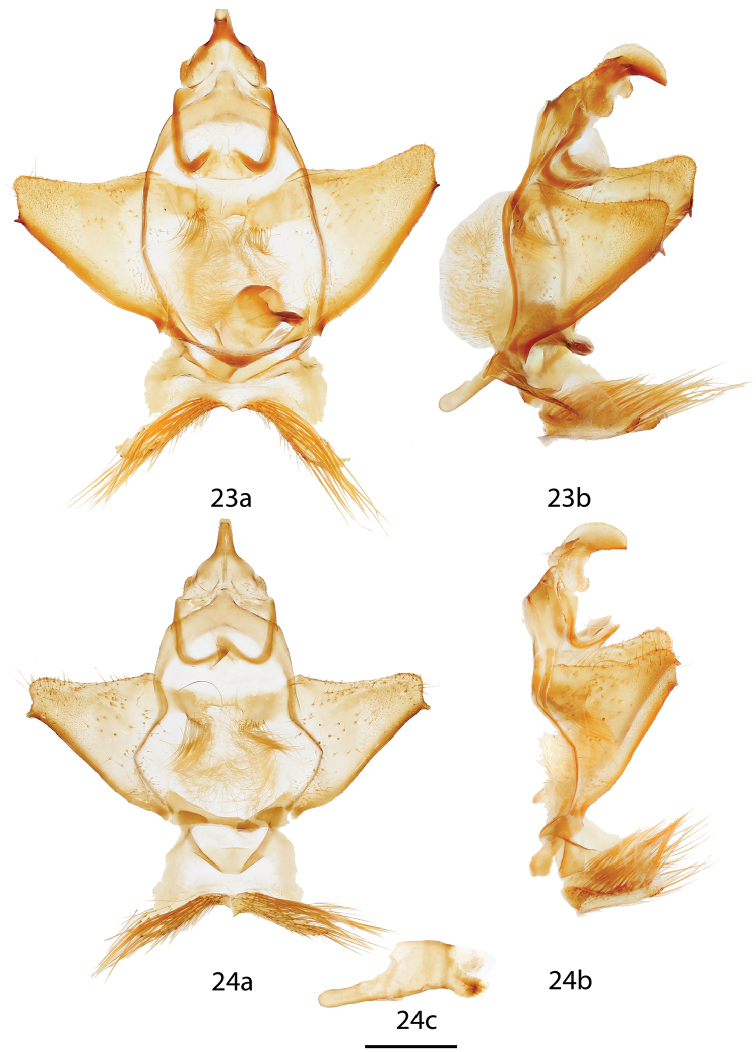
*Ulmara rotunda* ♂ genitalia, **a** ventral **b** lateral **c** phallus. **23** Lectotype, Colombia, Tolima, Nevado del Tolima, 3200 m, St Laurent diss.: 2-14-16:2 [phallus not excised] (USNM) **24** Colombia, Tolima, Nevado del Tolima, 3200 m, St Laurent diss.: 4-5-16:3 (MNHU). Scale bar = 1 mm.

**Figures 25–27.**
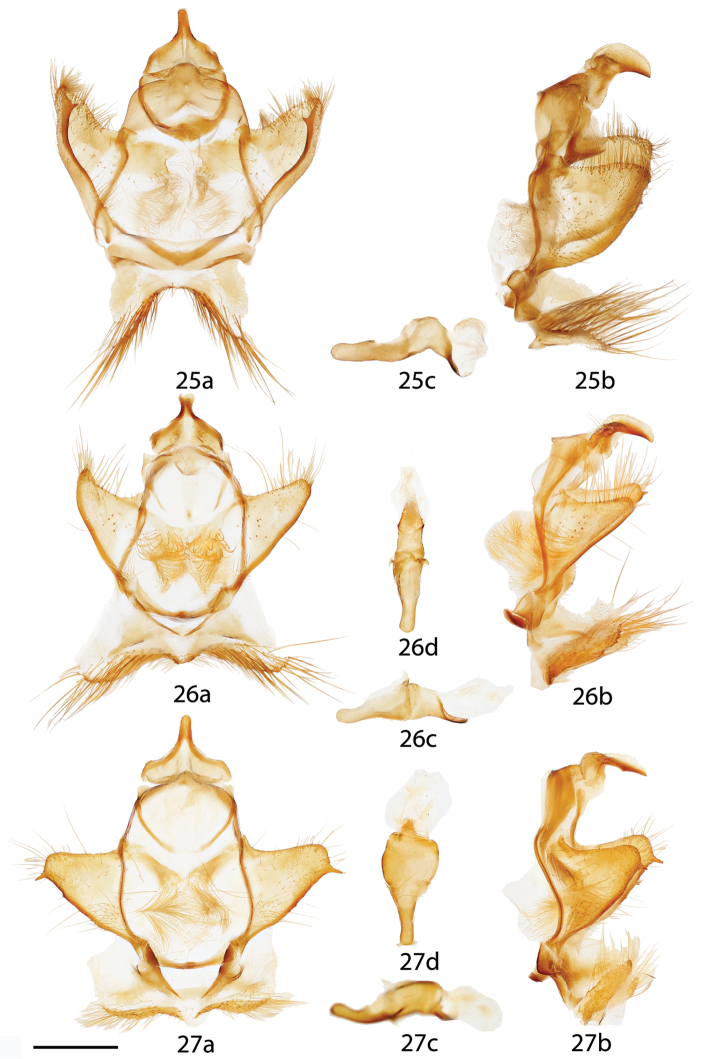
*Ulmara* ♂ genitalia, **a** ventral **b** lateral **c** phallus. **25** *Ulmara conjuncta* holotype ♂, Ecuador, Loja, N. Loja, road to Cuenca, 2220 m, St Laurent diss.: 4-5-16:4 [note: valves not fully spread in Fig. 25a] (CMNH) **26** *Ulmara azurula* holotype ♂, Peru, Huánuco, Carpish, 2700 m, St Laurent diss.: 4-8-16:2 (AMNH) **27** *Ulmara dombroskiei* holotype ♂, Peru, Puno, Carabaya, Agualani, 9000 ft., St Laurent diss.: 4-8-16:3 (NHMUK). Scale bar = 1 mm.

**Figure 28.**
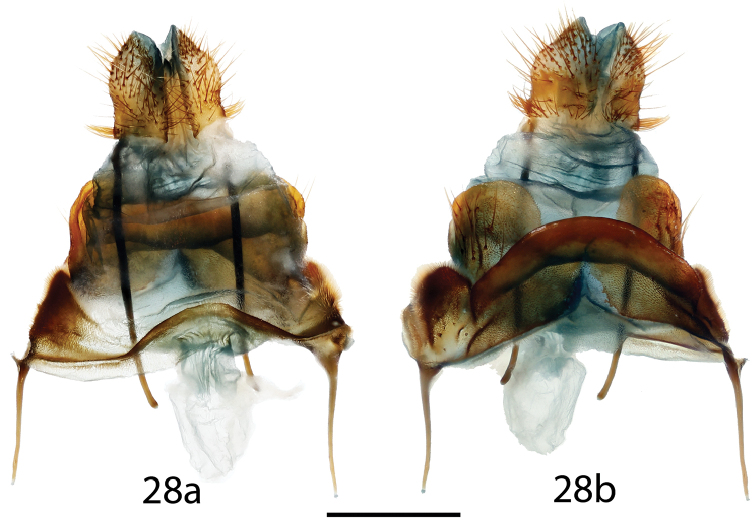
*Ulmara conjuncta* paratype ♀ genitalia, **a** ventral **b** dorsal. Ecuador, Loja, Road between Loja-Zamora, 2700 m, St Laurent diss.: 4-19-16:1 (MWM). Scale bar = 1 mm.

### Ulmara rotunda

Taxon classificationAnimaliaLepidopteraMimallonidae

(Dognin, 1916)

**Map 2.**
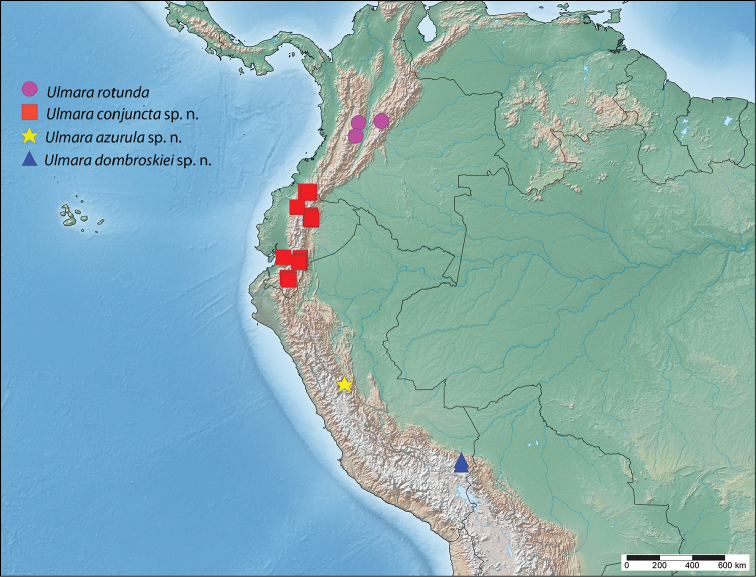
Known distribution of *Ulmara*.

Figs 14–16 , 23 , 24 ; Map 2

Cicinnus rotunda Dognin, 1916: 20–21
Ulmara rotunda ; Schaus 1928: Figs ♂ and ♀ 88 g
Ulmara rotunda ; Gaede 1931
Ulmara rotunda ; Becker 1996

#### Type material.

**2** ♂ **syntypes**, [deposition of one syntype unclear, but probably among series from type locality at MNHU or specimen “2602” in possession of R. Hutchings, available syntype from USNM designated as lectotype below]. Type locality: Colombia: Monte Tolima [Nevado del Tolima?].

#### Lectotype

**(here designated)**, ♂. **COLOMBIA: Tolima**: Monte Tolima, 3200 m, Colomb. Cent. Cord. [Nevado del Tolima?, Cordillera Central]/ Type No.: 29689 U.S.N.M./ USNM-Mimal: 1109/ St Laurent diss.: 2-14-16:2/ LECTOTYPE ♂ *Cicinnus rotunda* designated by St Laurent, 2016 [handwritten red label]/ (USNM).

#### Additional specimens examined.

(6 ♂, 1 ♀) **COLOMBIA: Tolima**: 3 ♂, Monte Tolima [Nevado del Tolima], 3200 m: Coll. Fassl, St Laurent diss.: 4-5-16:3 (MNHU). 1 ♂, Mt. Tolima [Nevado del Tolima], 2800 m: II.1910, A. H. Fassl, NHMUK010355070 (NHMUK). 1 ♂, San Antonio, 5800 ft: G.M. Palmer, St Laurent diss.: 4-15-16:1, NHMUK010355069 (NHMUK). **Cundinamarca/Distrito Capital**: 1 ♂, Bogotá: Rothschild Bequest BM 1939–1, St Laurent diss.: 4-15-16:2, NHMUK010355068 (NHMUK). 1 ♀, Bogotá [additional locality information illegible]: 13.IV.1871, Nolcken, Coll. Staudinger, “*Ulmara rotunda* ♀ type, Schaus” with label “Typus” [Not a true type, Schaus invalidly designated this as an allotype retroactively well after original description. Abdomen missing, no genitalia preparation.] (MNHU).

#### Diagnosis.

*Ulmara rotunda* is the largest and most northernmost representative of the genus, being the only species so far reported from Colombia. Apart from distribution and large size, *Ulmara rotunda* can be recognized by the nearly complete gnathos, which while not fused mesally, is only separated by a small gap with heavily sclerotized terminal ends on either side of the gap. The valves are among the largest in the genus, and are triangular, the phallus is also recognizable by its oblong shape where the distal half is much wider (when viewed laterally) than the basal half.

#### Description.

**Male.** *Head*: As for genus but labial palpus particularly long, extending well beyond frons. *Thorax*: As for genus. *Legs*: As for genus. *Forewing dorsum*: Forewing length: 19.05–21 mm, avg.: 20.4 mm, wingspan: 39–43 mm, n=6. As for genus but ground color dark brown rather than black, postmedial line located about one third wing length away from wing margin, usually very obvious due to lighter brown edging along inner side without blue sheen, postmedial line only weakly notched between Rs4 and costa. *Forewing ventrum*: As for genus, light-brown edging of postmedial line absent. *Hindwing dorsum*: Coloration as for forewing dorsum, antemedial line absent, discal mark absent, outer margin of wing serrate. *Hindwing ventrum*: As for genus. *Abdomen*: As for genus, but more robust, dorsally with lighter brown scales and well-defined dark terminus due to elongated tuft of dark-brown scales. *Genitalia*: (Figs 23, 24) n = 4. Vinculum ovoid, ventrally with reduced saccus, bilobed projections of saccus weakly attached to VIII, paired, setae-covered sclerotizations of VIII splayed mesally, extensions wider than ventral margin of vinculum. Uncus simple, highly truncated apically with triangular base. Gnathos nearly complete but gap present mesally, strongly sclerotized, especially terminally at gap. Valves moderately sized, triangular, somewhat truncated apically, with single tooth projecting from saccular edge near apex of valve, tooth variable in length. Transtilla with weakly sclerotized, inward facing setae-covered extensions. Setae pointed outward directly over phallus. Diaphragm forms large, balloon-like sac expanded inward into abdomen, sac covered in fine, inward facing setae surrounding phallus. Juxta partially fused to phallus, encircling it, lightly sclerotized, especially dorsal to phallus. Phallus short, stout, anterior half cylindrical, ventral apex heavily sclerotized, forming scoop-like extension below vesica; vesica small, sac-like, weakly scobinate; base of phallus much narrower than apical half, in same plane as apical half, not bent. **Female.** [description based on single, faded specimen] *Head*: As for male but antennae smaller overall, pectinations particularly shorter, labial palpus slightly reduced. *Thorax*: As for male. *Legs*: As for male, but vestiture seemingly sparser. *Forewing dorsum*: Forewing length: 21.5 mm, wingspan: ~41 mm, n = 1. As for male but more elongated, slightly narrower, discal mark reduced. *Forewing ventrum*: Somewhat similar to dorsum but much lighter, grayer, maculation reduced; antemedial line absent, postmedial line less distinct. *Hindwing dorsum*: Coloration as for forewing dorsum, antemedial line absent. *Hindwing ventrum*: Following same pattern as forewing ventrum. *Abdomen and genitalia*: Absent from unique specimen.

#### Distribution

**(Map 2).** *Ulmara rotunda* so far appears to be isolated within central Colombia with no records from neighboring countries. This species can be encountered at elevations ranging from 1767–3500 m.

#### Remarks.

Until the present work, only *Ulmara rotunda* was known from the genus, and was previously reported from both Nevado del Tolima and Bogotá (Schaus 1928), thus the only new location reported herein is San Antonio, Tolima, Colombia (NHMUK).

### Ulmara conjuncta

Taxon classificationAnimaliaLepidopteraMimallonidae

St Laurent sp. n.

http://zoobank.org/EC14FACE-6B67-4708-8E43-F99B4D12469C

Figs 17–19 , 25 , 28 ; Map 2

Ulmara rotunda ; Lemaire and Minet 1998: Fig. ♂ 18.2 B [Ulmara conjuncta sp. n. figured]

#### Type material.

**Holotype**, ♂. **ECUADOR: Loja**: ECUADOR: Loja. N Loja, road to Cuenca, 2220 m, 25 March 1993, Jan Hillman. Disturbed forest and pastures/ St Laurent diss.: 4-5-16:4/ HOLOTYPE ♂ *Ulmara conjuncta* St Laurent, 2016 [handwritten red label] (CMNH).

**Paratypes**, 23 ♂, 1 ♀. **ECUADOR: Carchi**: 2 ♂, El Angel Ecol. Reserve, road Tulcan-El Chical, 0°48'46"N, 78°00'40"W, 3300 m: 14.XI.2012, leg. Victor Sinyaev, Expedition Ron Brechlin (MWM). 2 ♂, 70 km Road El Chical-Tulcan, 0°50'29"N, 78°03'25"W, 2440 m: 22.XI.2012, leg. Sinyaev & Romanov, expedition Ron Brechlin (MWM). 5 ♂, El Angel Ecol. Reserve, 0°46'14"N, 78°03'27"W, 2785 m: 9–11.XI.2012, leg. Victor Sinyaev, expedition Ron Brechlin (MWM). 1 ♂, Road El Chical to Carolinae, 0°50'20"N, 78°13'39"W, 2360 m: 20.XI.2012, leg. Sinyaev & Romanov, Expedition Ron Brechlin (MWM). **Azuay**: 2 ♂, Cochabamba, Pan de Agucar, 2°46'18"S, 79°26'52"W, 2840 m: 11.XII.2012, leg. Sinyaev & Romanov, expedition Ron Brechlin (MWM). **Pichincha**: 2 ♂, Camping Bella Vista, 0°00'41"S, 78°41'17"W, 2230 m: leg. V. Siniaev & Romanov (MWM). **Napo**: 1 ♂, Cordillera Huacamayos [Cordillera Guacamayos], Estero Chico, virgin humid forest, 2650 m: 5.VIII.1996, J. Hillman, St Laurent diss.: 4-8-16:1 (CMNH). 1 ♂, Cordillera Huacamayos [Cordillera Guacamayos], San Isidro, Rio Aliso, 00-37-36S, 77-57-12W, deforested hacienda, 2250 m: 3.VIII.1996, Jan Hillman (CMNH). 1 ♂, Cordillera Huacamayos [Cordillera Guacamayos], San Fernando de Sierra Azul, disturbed montane forest, 2350 m: 4.VIII.1996, Jan Hillman (CMNH). 1 ♂, Rte Pifo to Baeza, km 52, 2600 m: 2–3.II.1975, H. Descimon, C. Herbulot, C. Lemaire, P. Thiaucourt, N. Venedictoff, Brit. Mus. 1975–140, *Ulmara rotunda* Dognin det. J.E. Chainey, 2003, NHMUK010355071 (NHMUK). 1 ♂, 6 km SE of Cosanga, 0°37'14"S, 77°54'08"W, 2240 m: 22.I.2012, leg. R. Brechlin & Siniaev (MWM). **Morona-Santiago**: 1 ♂, Rte Gualaceo to Méndez, km 41, 2400 m: 10–11.I.1975, H. Descimon, C. Herbulot, C. Lemaire, P. Thiaucourt, N. Venedictoff, St Laurent diss.: 4-15-16:3, Brit. Mus. 1975–140, *Ulmara rotunda* Dognin det. J.E. Chainey, 2003, NHMUK010355072 (NHMUK). 1 ♂, 9 km W Plan de Milagro to Gualaceo, 3°00'04"S, 78°30'49"W, 2375 m: 6–7.III.2013, leg. Ackermann, Käch & Dr. R. Brechlin (MWM). 2 ♂, Road Gualaceo-Plan de Milagro, 3°01'24"S, 78°35'06"W, 2157 m: 21.XI.2011, leg. V. Siniaev & O. Romanov (MWM). **Loja**: 1 ♀, Road between Loja-Zamora, 3°58'45"S, 79°08'28"W, 2700 m: 22.II.2012, leg. R. Brechlin & V. Siniaev, St Laurent diss.: 4-19-16:1 (MWM). – All paratypes with the following yellow label: PARATYPE ♂/♀ *Ulmara conjuncta* St Laurent, 2016.

#### Diagnosis.

This new species is most similar to *Ulmara rotunda* in external appearance, but can be distinguished from it by the more southerly distribution, darker, nearly black ground color, smaller size overall, lack of a brown inner edging along the postmedial line of the fore and hindwings, and the near absence of the postmedial line on the ventrum of the wings. The male genitalia also offer good characters for differentiation: in *Ulmara conjuncta* the gnathos is fused mesally, giving rise to a thin plate-like extension, smaller, more rounded valves, and the sharply downwardly bent phallus, which is much thinner overall and more elongated at the ventral apex. Other characters, such as the setae-covered sclerotizations on the ventrum of VIII, also differ, but are less readily useful in recognizing *Ulmara conjuncta*.

#### Description.

**Male.** *Head*: As for genus. *Thorax*: As for genus. *Legs*: As for genus. *Forewing dorsum*: Forewing length: 19–19.5 mm, avg.: 19.3 mm, wingspan: 36–37 mm, n=5. As for genus, except ground color nearly black, postmedial line located about one-third wing length away from wing margin, line usually obscured due to dark surrounding ground color and absence of lighter brown edging along inner side, overall with faint metallic-blue sheen, especially antemedially; postmedial line strongly notched between Rs4 and costa. *Forewing ventrum*: As for genus. *Hindwing dorsum*: Coloration as for forewing dorsum, antemedial line absent, discal mark absent, outer margin of wing weakly serrate. *Hindwing ventrum*: As for genus. *Abdomen*: As for genus, but slightly darker and less robust. *Genitalia*: (Fig. 25) n= 3. Vinculum somewhat box-like, ventrally with reduced saccus; saccus weakly attached to VIII, forming paired, setae-covered, downwardly-angled sclerotizations, not splayed beyond width of vinculum. Uncus simple, highly truncated apically with wide triangular base. Gnathos fused, extended mesally as thin plate that may be notched mesally. Valves small, subtriangular, edges somewhat curled, with single tooth projecting from saccular edge near apex of valve, tooth variable in length. Transtilla with weakly-sclerotized, inward-facing setae-covered extensions; setae very fine. Setae pointed outward directly over phallus. Diaphragm forms small balloon-like sac expanded inward into abdomen; sac covered in fine, inward-facing setae surrounding phallus. Phallus short, anterior half moderately to sharply bent downward, ventral apex heavily sclerotized into elongated point; vesica small, sac-like, weakly scobinate with some evidence of diverticula; base of phallus narrower than apical half, in same plane as apical half, not bent. **Female.** *Head*: As for male but antennae much smaller overall, pectinations particularly shorter, labial palpus slightly reduced. *Thorax*: As for male. *Legs*: As for male, but vestiture thinner. *Forewing dorsum*: Forewing length: 21.5 mm, avg.: 21.5 mm, wingspan: 41 mm, n= 1. As for male, but more elongated, slightly narrower, discal mark reduced. *Forewing ventrum*: Somewhat similar to dorsum but much lighter, grayer, maculation reduced; antemedial line absent, postmedial line less distinct. *Hindwing dorsum*: Coloration as for forewing dorsum, antemedial line absent. *Hindwing ventrum*: Following same pattern as forewing ventrum. Frenulum as multiple bristles. *Abdomen*: See generic description. *Genitalia*: See generic description.

#### Distribution

**(Map 2).** This species is known from northern and southern Ecuador, with records lacking from the center of the country. *Ulmara conjuncta* is another Andean species, and is found at elevations ranging from 2157–3300 m.

#### Etymology.

This species is named for the complete, connected (*conjuncta* Latin), gnathos, a character unique to this species in the genus.

#### Remarks.

*Ulmara conjuncta*, although similar to *Ulmara rotunda*, is easily distinguished by both distribution and all characters given in the diagnosis. According to the data on the labels of a number of specimens collected by Jan Hillman, this species can be collected in “virgin humid forests” as well as “disturbed” forests, pastures, and “deforested haciendas.”

This is the only species in the genus with a well-defined gnathos, which, when present, is an important character for distinguishing similar species within genera in Mimallonidae (pers. obs).

### Ulmara azurula

Taxon classificationAnimaliaLepidopteraMimallonidae

St Laurent sp. n.

http://zoobank.org/3B1C65B5-CDC0-4628-9C32-0C8289587A45

Figs 20 , 26 ; Map 2

#### Type material.

**Holotype**, ♂. **PERU: Huánuco**: Carpish, Huanuco [Huánuco], Peru 2700 m, Oct. 25, 1946/ Felix Woytkowski Collector/ St Laurent diss.: 4-8-16:2/ HOLOTYPE ♂ *Ulmara azurula* St Laurent, 2016 [handwritten red label] (AMNH).

**Paratypes**, 4 ♂. **PERU: Huánuco**: Carpish, 2700 m: 8.X.1946 (2 ♂), 14.X.1946 (1 ♂), 26.X.1946 (1 ♂), Felix Woytkowski Collector, St Laurent diss.: 4-5-16:5 (AMNH). – All paratypes with the following yellow label: PARATYPE ♂ *Ulmara azurula* St Laurent, 2016.

#### Diagnosis.

*Ulmara azurula* is the smallest species in the genus (on average), and so far the only species known from central Peru. The small size and distribution should allow this species to be distinguished from the previous two. The following characters differentiate *Ulmara azurula* from the most similar species, *Ulmara dombroskiei* sp. n. to be described below, but also differentiate it from both *Ulmara rotunda* and *Ulmara conjuncta*. Although *Ulmara azurula* is externally similar in size and coloration to *Ulmara dombroskiei* sp. n., namely by the metallic bluish sheen, in *Ulmara azurula*, the postmedial line of the fore and hindwings is located closer to the wing margin than in *Ulmara dombroskiei* sp. n. Furthermore, the width of the splayed, setae-covered sclerotizations of VIII are much wider than the vinculum, whereas in *Ulmara dombroskiei* sp. n. these sclerotizations are highly reduced overall, and have shorter setae. Additionally, the gnathos of *Ulmara azurula* is the most reduced of the entire genus, and exists as only weakly-sclerotized lateral bars, whereas in *Ulmara dombroskiei* sp. n. the gnathos nearly converges mesally. The setae-covered flaps projecting inward from the transtilla in *Ulmara azurula* bear thicker setae than in any other *Ulmara*. Finally, the phallus shape is perhaps the best genital character for distinguishing these two Peruvian species: in *Ulmara azurula* the phallus is uniformly cylindrical from a dorsal perspective, whereas in *Ulmara dombroskiei* sp. n. the phallus is very wide distally.

#### Description.

**Male.** *Head*: As for genus. *Thorax*: As for genus. *Legs*: As for genus. *Forewing dorsum*: Forewing length: 17.5–19 mm, avg.: 18.1 mm, wingspan: 35–36 mm (approximate), n= 5. As for genus but ground color nearly black, overall metallic-blue sheen present due to angled, reflective scales. Postmedial line about one-fourth wing length away from wing margin, postmedial line obvious due to pale blue-gray suffusion surrounding line, postmedial line nearly straight between Rs4 and costa. *Forewing ventrum*: As for genus. *Hindwing dorsum*: Coloration as for forewing dorsum, antemedial line absent, discal mark absent, outer margin of wing weakly serrate. *Hindwing ventrum*: As for genus. *Abdomen*: As for genus. *Genitalia*: (Fig. 26) n=2. Vinculum somewhat box-like, ventrally with reduced saccus, bilobed saccus attached to VIII, forming paired, setae-covered sclerotizations; splayed sclerotizations wider than ventral margin of vinculum. Uncus simple, highly truncated apically with wide base. Gnathos highly reduced, with only weakly sclerotized lateral arms. Valves small, subtriangular, with single tooth projecting from saccular edge near apex of valve, tooth variable in length. Transtilla with weakly-sclerotized, inward-facing setae-covered extensions; setae very thick. Setae pointed outward directly over phallus. Diaphragm forms small balloon-like sac expanded inward into abdomen, sac covered in fine, inward-facing setae surrounding phallus. Juxta partially fused to phallus, encircling it, lightly sclerotized, especially dorsal to phallus. Phallus short, anterior half cylindrical, straight, ventral apex heavily sclerotized as elongated, sharp point, vesica small, sac-like, weakly scobinate; base of phallus narrower than apical half, basal part shorter than, and in same plane, as cylindrical terminal part. **Female.** Unknown.

#### Distribution

**(Map 2).** This new species is known only from the type locality in Huánuco, central Peru, at an elevation of 2700 m.

#### Etymology.

This species is named for its small size (diminutive –*ula* Latin) and blue (*azurea* Latin) iridescence.

#### Remarks.

Although the known range of this species is located between the distributions of *Ulmara conjuncta* in Ecuador and *Ulmara dombroskiei* sp. n. in southern Peru, *Ulmara azurula* is not intermediate in external or genital morphology between these species, but is instead more similar to *Ulmara dombroskiei* sp. n. than either previously described species. Considering the consistently smaller size, almost entirely absent gnathos, narrower phallus, and more elongated saccular extensions relative to *Ulmara dombroskiei* sp. n., as well as previously mentioned wing characters, this species is apparently distinct from the southern Peruvian populations named *Ulmara dombroskiei* sp. n. below. The iridescent blue sheen, characteristic of the genus *Ulmara*, is most distinct in the Peruvian species, but is not clearly reproduced in photographs.

### Ulmara dombroskiei

Taxon classificationAnimaliaLepidopteraMimallonidae

St Laurent sp. n.

http://zoobank.org/4FFDF534-9C02-41C2-906C-654A98E1F7BF

Figs 21 , 22 , 27 ; Map 2

#### Type material.

**Holotype**, ♂. **PERU: Puno**: Agualani, Carabaya [Puno], 9000 ft., Dec. 05. wet season, (G.R. Ockenden)./ *Ulmara rotunda* Dognin, Pearson det./ Rothschild Bequest BM 1939–1/ NHMUK010355067/ St Laurent diss.: 4-8-16:3/ HOLOTYPE ♂ *Ulmara dombroskiei* St Laurent, 2016 [handwritten red label]/ (NHMUK).

**Paratypes**, 4 ♂. **PERU: Puno**: 2 ♂, Santo Domingo, Carabaya [Puno], 6000 ft: III.1902, IV.1902, Ockenden, wet season and end of wet season, Rothschild Bequest BM 1939–1, St Laurent diss.: 4-15-16:4, NHMUK010355073, 010354877 (NHMUK). 1 ♂, Santo Domingo, S. E. Peru, 6000 ft: G. Ockenden, “not in B. M.,” Joicey Coll. Brit. Mus. 1925–157, NHMUK010354878 (NHMUK). 1 ♂, Santo Domingo, Carabaya [Puno], 6500 ft: XII.1902, G. Ockenden, wet season, 623, Rothschild Bequest BM 1939–1, NHMUK010355066, St Laurent diss.: 4-26-16:1 (NHMUK). – All paratypes with the following yellow label: PARATYPE ♂ *Ulmara dombroskiei* St Laurent, 2016.

#### Diagnosis.

This species is most similar to *Ulmara azurula*, see the diagnosis of that species for characters necessary for differentiation. However, it is worth repeating the characters entirely unique to this species in the entire genus: postmedial line somewhat variable in distance from wing margin but may be as far as one-third wing length away from the margin, the gnathos is reduced but nearly converges mesally (or does so very weakly), and is overall not heavily sclerotized, distinguishing it *Ulmara rotunda*, the uncus is highly truncated, the saccular extensions are highly reduced, and the phallus is very wide distally.

#### Description.

**Male.** *Head*: As for genus. *Thorax*: As for genus. *Legs*: As for genus. *Forewing dorsum*: Forewing length: 17.5–19.5 mm, avg.: 18.7 mm, wingspan: 34–37 mm, n= 3. As for genus but ground color nearly blue-gray, overall blue metallic sheen present due to angled, reflective scales. Postmedial line located about one-fourth to one third wing length distant from wing margin, postmedial line obvious due to pale blue-gray suffusion surrounding line, postmedial line sharply notched between Rs4 and costa. *Forewing ventrum*: As for genus. *Hindwing dorsum*: Coloration as for forewing dorsum, antemedial line absent, discal mark absent, outer margin of wing weakly to moderately serrate. *Hindwing ventrum*: As for genus. *Abdomen*: As for genus. *Genitalia*: (Fig. 27) n=3. Vinculum somewhat box-like, ventrally with reduced saccus, bilobed projections of saccus attached to VIII, forming small, paired, setae-covered sclerotizations, splayed sclerotizations barely extending beyond width of vinculum. Uncus simple, highly truncated apically with very wide base. Gnathos incomplete, reduced, but nearly converging mesally, weakly sclerotized. Valves small, subtriangular, with single tooth projecting from saccular edge near apex of valve, tooth variable in length. Transtilla with weakly-sclerotized, inward-facing setae-covered extensions, setae very fine. Setae pointed outward directly over phallus. Diaphragm forms small balloon-like sac expanded inward into abdomen, sac covered in fine, inward facing setae surrounding phallus. Phallus short, anterior half greatly widened, ventral apex more heavily sclerotized forming point, vesica small, sac-like, weakly scobinate; base of phallus much narrower than apical half, basal part much narrower than terminal part, angled slightly downward. **Female**: Unknown.

#### Distribution

**(Map 2).** This species is known only from two nearby localities, separated by about 30 km, in Puno, southeastern Peru at elevations ranging from 1828–2743 m, and is the most southerly distributed species of *Ulmara*.

#### Etymology.

This species is named for Jason Dombroskie (CUIC), a colleague, mentor, and friend to the author. Additionally, Jason is one of the few individuals who has been involved in the taxonomy of the poorly known Mimallonidae, and thus has been integral in deepening our understanding of this fascinating family of moths.

#### Remarks.

As previously mentioned in the remarks for *Ulmara azurula*, the two Peruvian species, although distinct, are related, given the reduction of the gnathos, the small valves, and the blue iridescence of the wing scales. As mentioned previously, this blue iridescence is not well represented in the figures.

Although five specimens and three dissections of *Ulmara dombroskiei* were examined when writing the description, only one specimen (the holotype) is from Agualani at the higher elevation. This specimen displayed the most accentuated characters that define this species, namely the very broad phallus and the postmedial lines of the fore and hindwings, which are relatively distant from the wing margin. Therefore, this specimen is designated as the holotype. The differences between the holotype and paratypes, namely slightly less broadened phallus and postmedial line located nearer to the wing margin, may be due to elevation differences considering the proximity of the localities and the otherwise same general structures of the genitalia.

### Cunicumara

Taxon classificationAnimaliaLepidopteraMimallonidae

St Laurent gen. n.

http://zoobank.org/B4788F32-4454-49E0-AFFD-A93E144B9849

#### Type species.

*Cunicumara anae* St Laurent, 2016, sp. n.

#### Etymology.

The name for this new genus comes from *cuniculus* (Latin), meaning rabbit, referring to the somewhat layered appearance of brown coloration of *Cunicumara anae* sp. n., which is reminiscent of rabbit fur. Furthermore, the particularly long antennae relative to the size of the body and wings is quite remarkable, therefore the name is appropriate in calling to mind the long ears of a rabbit. The ending –*mara* notes a slightly superficial similarity to *Ulmara*.

#### Diagnosis.

This new genus can be recognized by the salmon to orange-brown, fading to light, sandy tan ground color, interspersed with gray, pale-khaki, and dark-brown scales, which give moths of this genus a somewhat hoary appearance. The wings are very broad, with a weakly accentuated, barely falcate apex. The long bipectinate antennae are more than half the length of the short forewings, and have distinctly long pectinations. The combination of these characters should immediately allow *Cunicumara* to be distinguished from all other known Mimallonidae. The complex male genitalia are also unique, characterized by the presence of a basally-fused, bifurcated gnathos, basally-toothed sacculus, and the phallus with curled, horn-like juxtal processes, which are dorsal to the phallus; a third, singular horn-like process arises between the curled juxtal processes .

#### Description.

**Male.** *Head*: Khaki brown, eyes very large, occupying more than two-thirds area of head, bordered posteriorly by dark scales; antenna coloration pale tan, very long, more than half length of forewing, antenna bipectinate to tip, pectinations very long, longest nearly one-fourth length of antennae overall; labial palpus very reduced, not extending beyond head, apparently three segmented, but segmentation obscured by thick tufts ventrally. *Thorax*: Coloration as for head but with gray-tipped scales giving thorax a hoary appearance overall. *Legs*: Coloration as for thorax, vestiture thick, long. Tibial spurs robust, covered in scales except for tip. *Forewing dorsum*: Forewing length: 14–15.5 mm, avg.: 14.8 mm, wingspan: 30–31 mm, n=3. Short, subtriangular, margin convex except for where concave very near apex; apex slightly falcate. Ground color light with slight salmon hue, especially along anterior half of wing until costa; wing with layered appearance due to presence of light-gray, dark-brown, and brown scattered scales, particularly contrasting gray scales concentrated basally and along costa, giving wing hoary appearance. Antemedial line very faint, light brown, somewhat wavy. Gray-brown postmedial line straight from anal margin until Rs4 where line angled toward costa. Antemedial, medial, and postmedial areas mostly concolorous, although ante- and medial areas appear lighter due to gray scales; likewise salmon and light-brown coloration of ground color most evident near apical angle of postmedial line and postmedially. Discal mark nearly absent, or as thick, dark streak spanning cell. Fringe poorly preserved in examined specimens, but consisting of elongated scales, coloration as for medial area except some lighter off-white scales present in semi-regular pattern along margin. *Forewing ventrum*: Very similar to dorsum but appearing lighter, due to more diffuse presence of gray scaling, dark-brown scales may also be more prevalent in some individuals; antemedial line absent, postmedial line less distinct, outwardly curved, roughly following wing margin in shape, not straight as on dorsum. *Hindwing dorsum*: Coloration, patterning as for forewing dorsum, but antemedial line absent, discal mark absent, postmedial line slightly curved following outline of wing margin, postmedial line may be very faint. *Hindwing ventrum*: Following same pattern as forewing ventrum, but lighter due to pale-khaki scales basally. Frenulum present as single bristle. *Venation*: More typical of Mimallonidae than either *Lurama* or *Ulmara* with respect to M_1_–M_3_, with each vein being more evenly spaced along obliquely angled cell, CuA_1_ arises from lower angle of cell; Rs3 + Rs4 short stalked. *Abdomen*: Lighter than thorax, with coppery luster in fresh specimen, fading to pale khaki in older material. Vestiture thick, long, distal tip of abdomen with elongated, dark-brown tipped scales. *Genitalia*: (Fig. 31) n= 2. Complex. Vinculum somewhat ovoid, ventrally with reduced rounded saccus. Uncus simple, triangular, truncated apically to point. Gnathos very robust, broad basally arising from heavy sclerotization of tegumen, gnathos separated distally into paired, fingerlike processes. Valves elongated, narrow, weakly curved distally, sacculus with prominent, heavily sclerotized tooth. Mesal costal ridge present along basal half of valve. Juxta partially fused to phallus, juxtal processes present dorsal to phallus, processes curled forming shape of bovid horns, between processes arises a third, somewhat flattened, singular process, unlike curled processes on either side; strong membrane connects juxtal processes to base of vinculum, ventral lip of juxta fused to vinculum base, lip cut to excise phallus. Base of phallus with somewhat elongated backward-facing lobes. Phallus short, broadened distally, width of phallus somewhat variable, vesica short, bag-like. **Female.** Unknown.

#### Remarks.

*Cunicumara* is distinct among the family Mimallonidae, externally bearing some resemblance to *Ulmara* by the presence of the massive antennae and the short, broad wings, while at the same time displaying male genitalia that in some ways resemble the distinct genus *Menevia* Schaus, 1928, namely the presence of complex juxtal processes and the basally bifurcated lobes of the phallus.

### Cunicumara anae

Taxon classificationAnimaliaLepidopteraMimallonidae

St Laurent sp. n.

http://zoobank.org/A5E187EE-7AC8-4E23-AC35-7156DA0188F3

**Map 3.**
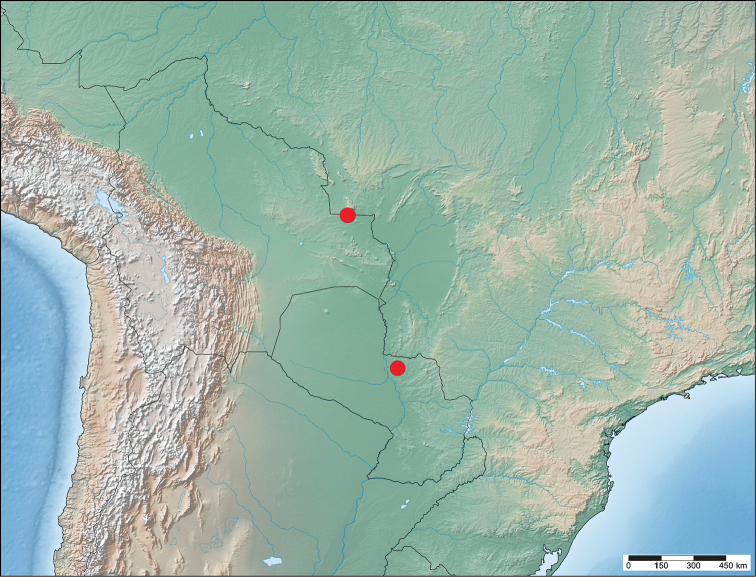
Known distribution of *Cunicumara anae*.

**Figures 29, 30.**
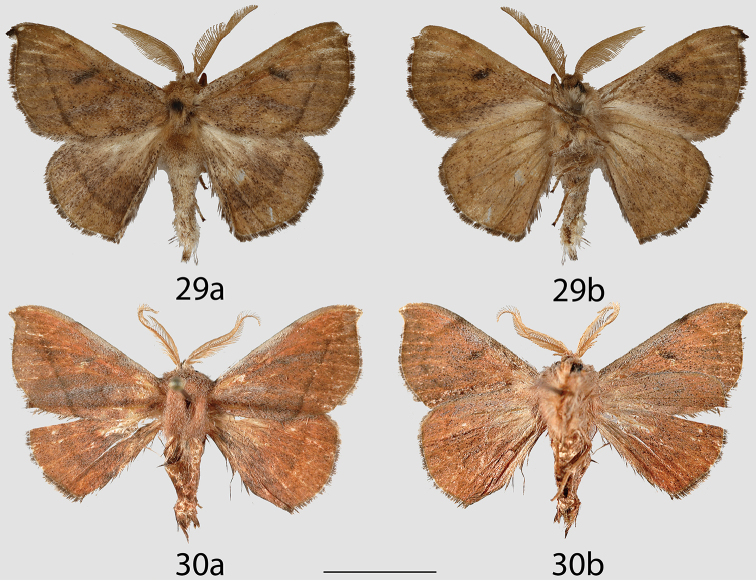
*Cunicumara anae* adults, **a** dorsal **b** ventral. **29** Holotype ♂, Bolivia, Santa Cruz, Asunción (CMNH) **30** Paratype ♂, Paraguay, Concepción, Garay Cue, 212 m [photo courtesy of Daniel Herbin] (CDH). Scale bar = 1 cm.

**Figure 31.**
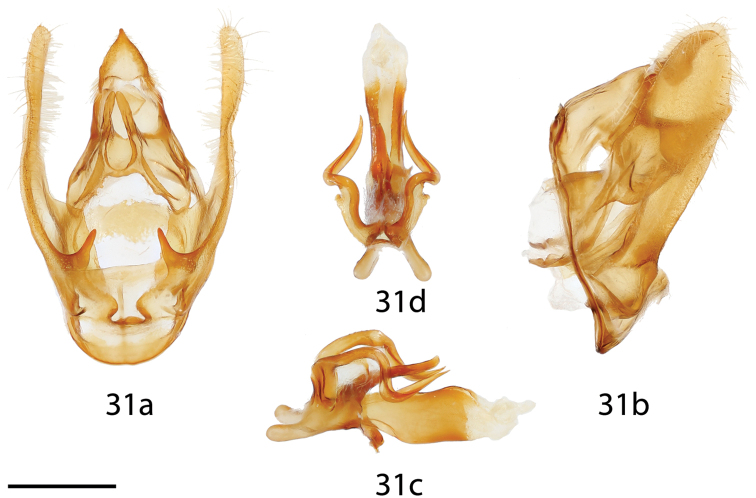
*Cunicumara anae* holotype ♂ genitalia, **a** ventral **b** lateral **c** phallus lateral **d** phallus dorsal. Bolivia, Santa Cruz, Asunción, St Laurent diss.: 3-14-16:6 [note: valves not fully spread in Fig. 31a] (CMNH). Scale bar = 1 mm.

Figs 29–30 , 31 ; Map 3

#### Type material.

**Holotype**, ♂. **BOLIVIA: Santa Cruz**: Asumpcion [Asunción], Bolivia, June 15, 1909, Haseman/ Carn. Mus., Acc. 4043/ Asumpsion, Bolivia, June 15-1909/ St Laurent diss.: 3-14-16:6/ HOLOTYPE ♂, *Cunicumara anae* St Laurent 2016 [handwritten red label]/ (CMNH). Type locality: Bolivia: Santa Cruz: Asunción.

**Paratypes**, 2 ♂. **BOLIVIA: Santa Cruz**: 1 ♂, Asunción: 15.VI.1909, Haseman leg. (CMNH). **PARAGUAY: Concepción**: 1 ♂, Garay Cue, 22°41'S, 57°22'W, 212 m: 4–9.VI.2013, Coll. D. Herbin, BC-Her 4868 [BOLD barcode number], D. Herbin genitalia prep. H1105 (CDH). – Paratypes with the following yellow label: PARATYPE ♂ *Cunicumara anae* St Laurent, 2016.

#### Diagnosis.

See generic diagnosis.

#### Description.

See generic description.

#### Distribution

**(Map 1).** This species is known only from Santa Cruz Department, Bolivia, and Concepción Department, Paraguay at low elevations.

#### Etymology.

This species is named for Ana Paula dos Santos de Carvalho, a lepidopterist interested in the sexual dynamics of Papilionoidea. Ana is the best friend and source of the most joy in the author’s life.

#### Remarks.

This new species, and thus the genus *Cunicumara* in general, is very poorly represented in collections. However, very little Mimallonidae material is available from the type locality or similar regions in Brazil, Paraguay, and Bolivia, and thus the rarity of this species in collections is probably only due to under collecting. The two localities both border on the Brazilian Pantanal Biome (IBGE 2004), and thus it is possible that this species is endemic to the wet lowlands, or the borders thereof, of this part of South America.

The two figured specimens (Figs 29 and 30) differ in coloration and antenna size. However, it is critical to note that the Bolivian specimens were collected in 1909 while the Paraguayan specimen was collected much more recently, in 2013. Therefore, differences in color can be attributed to the wide range of dates of collection. The salmon color that is apparent on the fresh specimen is faint, but present, when examining the wings of the old specimens under magnification. Furthermore, the figured Bolivian specimen (the holotype) is missing the distalmost portion of the antennae. Genitalia of these specimens are nearly identical (St Laurent diss.: 3-14-16:6 and D. Herbin genitalia prep. H1105).

According to Daniel Herbin (pers. comm.) the COI barcode data for Paraguayan paratype (BC-Her 4868) is quite distinct from other genera.

## Supplementary Material

XML Treatment for Lurama

XML Treatment for Lurama penia

XML Treatment for Lurama quindiuna

XML Treatment for Ulmara

XML Treatment for Ulmara rotunda

XML Treatment for Ulmara conjuncta

XML Treatment for Ulmara azurula

XML Treatment for Ulmara dombroskiei

XML Treatment for Cunicumara

XML Treatment for Cunicumara anae
